# Occupational Health Center as a Novel Model of Health Care Organization in Tertiary Educational Setting

**DOI:** 10.3389/fpubh.2022.866223

**Published:** 2022-05-02

**Authors:** Sára Felszeghi

**Affiliations:** Healthcare and Methodology Centre of the Faculty of Health Sciences of the University of Miskolc, Miskolc, Hungary

**Keywords:** occupational health center, multidisciplinary healthcare, prevention, education, equal opportunities, work ability, extending the active life

## Abstract

The activity of the Occupational Health Center of the University of Miskolc was awarded the Best practice in Occupational Medicine by the European Network for Workplace Health Promotion in 2013. This study presents the model, which promotes multidisciplinary health service, combined with education and research as a novel element, and will outline its impact on national and international research.

## Introduction

The Occupational Health Center is part of the Healthcare and Methodology Center of the University of Miskolc. Miskolc, which is the third largest city of Hungary, lies in the north–eastern part of the country. The city used to be an industrial town in the past, but nowadays it is a university center. The University of Miskolc is a real “Universitas” with eight faculties as follows: Earth Science and Engineering, Materials Science and Engineering, Mechanical Engineering and Informatics, Law, Economics, Arts, Health Sciences, Music “Béla Bartók.” Around 9,000 students study in the university and it has more than 1,400 employees.

The campus of the University of Miskolc is situated in the outskirts of the city, in a greenbelt, called Egyetemváros in Hungarian (“City of the University” in English). Developing the Occupational Health Center meant a positive change as discussed in the following: The disadvantage of the location, being outside the city center, was turned into an advantage when the health services were brought closer to all university people.

## Foundation of the Center

In Hungary, the compulsory work ability examination for all contractual employees is granted by the Act XLIII 1993, Law of Health and Safety at Work (Munkavédelmi törvény in Hungarian) and regulated by the governmental and ministerial decrees 27/1995 on Occupational Health Care, 89/1995 on Occupational Health Service and 33/1998 on Physical and Professional Examination and Assessment of Professional and Personal Hygiene.

After having assessed the health status of the employees at the University of Miskolc in 1988 (at that time the Technical University of Heavy Industry), having analyzed the health risks (smoking, excessive alcohol consumption, stress, inadequate nutrition, and lack of exercise) and morbidity indicators, the author realized that a radical change was needed to protect the health of the workers and prevent diseases. Thus, in 1989, the author developed a complex health promotion program for university employees which encompassed all forms of prevention in addition to acute patient care, as well as early workplace rehabilitation and the care for chronic patients.

This program, which at that time was unusual, became the starting point for a model that changed the structure and professional content of occupational health care. It also became clear that the traditional structure used at that time in health care was not suitable for the implementation of this program. Since 1997, this model has been known as occupational health promotion and has been applied throughout Europe.

Therefore, in 1995, a complex, multidisciplinary clinic ensemble suitable was established suitable for this purpose, which became known as the Occupational Health Center (in Hungarian Foglalkozás Egészségügyi Központ, FEK). This innovative form of health care, which is the most modern and complex version of occupational health care both in Hungary and abroad, was the first such clinic complex in the country.

The development the Health Care Center has evolved into is establishing the Center for Health Care and Methodology. This is a multidisciplinary healthcare center, with basic and special occupational healthcare, general practice, family physician, dental health service, laboratory, ophthalmology, otolaryngology, audiology, neurology, rheumatology, physiotherapy, and gynecology. We also have a 25-bed in-patient section to provide health services for students.

The author's goal was to ensure equal opportunities for all university employees, regardless of the post they held. With the individual consent of the person, we would carry out a full examination of the employee, which is an important factor in early diagnosis. In 1995, this method was introduced for students as well.

## The Activity Areas of the Center

The Center for Healthcare and Methodology covers three main areas of activity as follows:

- **Complex health service:** Covers treatment, rehabilitation, continuous, and complex healthcare.
- **Education:** Involves employees and students at the university, residents, and postgraduate specialists in occupational medicine.
- **Research:** Focuses on different areas including aging employees, psychosocial risks, ergonomic risks, and workplace health promotion.

We started our first complex healthcare program The Duties and Possibilities of Occupational Healthcare Specialists in Extending the Active Lifespan of Employees in 1989 (1, 2). This program initially targeted the employees, and it has been extended to the students since 1995 including the activities targeting the possibilities and duties of the Occupational Healthcare Service.

Besides the annual revision and analysis, we also completed health check every 3–5–7 years and introduced the necessary corrections. In 2000, we introduced the ISO quality insurance system (3), involving the management, technical workers, teaching staff, and students as well as the occupational healthcare workers. In 2019, we introduced a new strategy to prevent COVID-19 infections and organized the rehabilitation of those suffering from it. This strategy was recommended by the ministry to all higher education institutions as a model.

## An Outline of the Program

### Risk Analysis

The risk factors are due to the work environment, the work activity, the workers' health condition, and the morbidity. As an important aspect, we introduced the individual risk analysis (4), and we carried out not only the working task related risk analysis but also the individual risk analysis for each employee.

- The work environment is controlled by periodical occupational hygiene inspections. The employee must have adequate conditions at work, and if any irregularity occurs, it is imperative to act immediately, which is a continuous duty.
- Working activity in our case includes the activity of employees of the university, teachers, and administrative staff.
- The work of administrative staff is a sedentary occupation; in most cases, these are computer-based workplaces. The teaching staff works 8–10 h per day, exceeding the official 6 h stipulated in the special law for computer-based workplaces.
- The following requires a special attention: The evaluation of the special ergonomic risk and increased stress is imperative.
  - Physical risks include lighting factors, temperature, noise level, etc.
  - Chemical risks are not typically encountered by administrative staff (this is only relevant only in the case of laboratory workers).
  - Biological risks are more frequently encountered by the employees of the Faculty of Health Sciences and due to the COVID-19 pandemic. Since all people involved with the university could be infected, we can ask the following question: “Is this an occupational disease?”

Psycho–social risk is an increasing factor in this environment; we meet the problem of attenuated stress–distress, time pressure, overloading, and uncertainty. The system of home office and online teaching also increases the level of psycho–social risk.

### Work Ability Examination

The work ability consultation is one of the main duties in the occupational health service (5).

It must include the family and workplace history and a declaration on hereditary diseases and disorders. The occupational diseases must be diagnosed and followed up in cooperation with the respective specialized national services. A special attention should be given to new challenges such as burnout, Monday morning sickness, etc. Lifestyle could be an important factor as well; the program did follow up two major elements: Smoking habit and alcohol abuse of employees.

The physical examination involving all risk factors such as blood pressure, blood sugar, and cholesterol levels etc. is an important part of the work ability consultation of the employee. More than 31% of chronic diseases are diagnosed at our center. The maximum validity of the examination is for 1 year. This systematic and periodic control ensures the upkeep continuous care.

Vaccination has been a special duty during the COVID-19 pandemic. Since January 2021 at our center, we vaccinated people in the following categories:

- We vaccinated 1,200 patients (first and second doses) and an additional 180 people (third dose) on voluntary basis.
- As of September 2021, vaccination against COVID-19 is mandatory for healthcare workers: Here we vaccinated 1,000 students (2,000 vaccination doses) and 50 teachers (100 vaccinations).

### Continuous Healthcare

**Figure 1:**
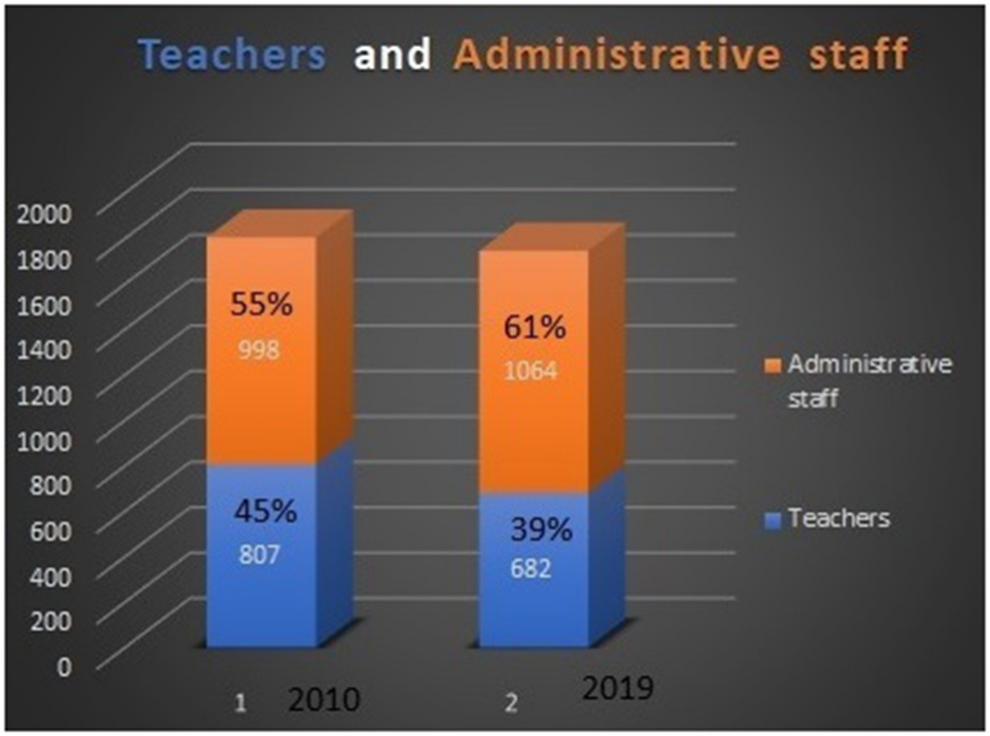
The ratio of teachers and administrative staff in the University of Miskolc.

Periodically, monthly or every 2 months, we check the health statuses according to the employee's ailment and their working conditions (see Figure 1).

We consider their workload and exposition to follow up on their health status based on the work ability index and we suggest any necessary improvements—in work-environment, working activity and healthcare, and in medication.

### Rehabilitation

It is a legal duty of the employer and occupational healthcare provider to ensure the workplace rehabilitation of the employee. The employer must create suitable work conditions for the employee with disabilities.

The occupational health specialist offers advice for the employer concerning the necessary workplace condition, and examines the work ability of the employee, suggesting any necessary restrictions to be implemented, or other work–activities in the case of temporary inabilities or permanent disabilities. In this case, the occupational health specialist proposes the retraining of the employee.

The key for successful rehabilitation is the full cooperation of the employee.

The University of Miskolc offers rehabilitation at the workplace in any of these forms necessary for the individual: Special medical gymnastics, swimming, yoga, physiotherapy, stress–management techniques, and flexible working hours if needed.

### Workplace Health Promotion

**Figure 2:**
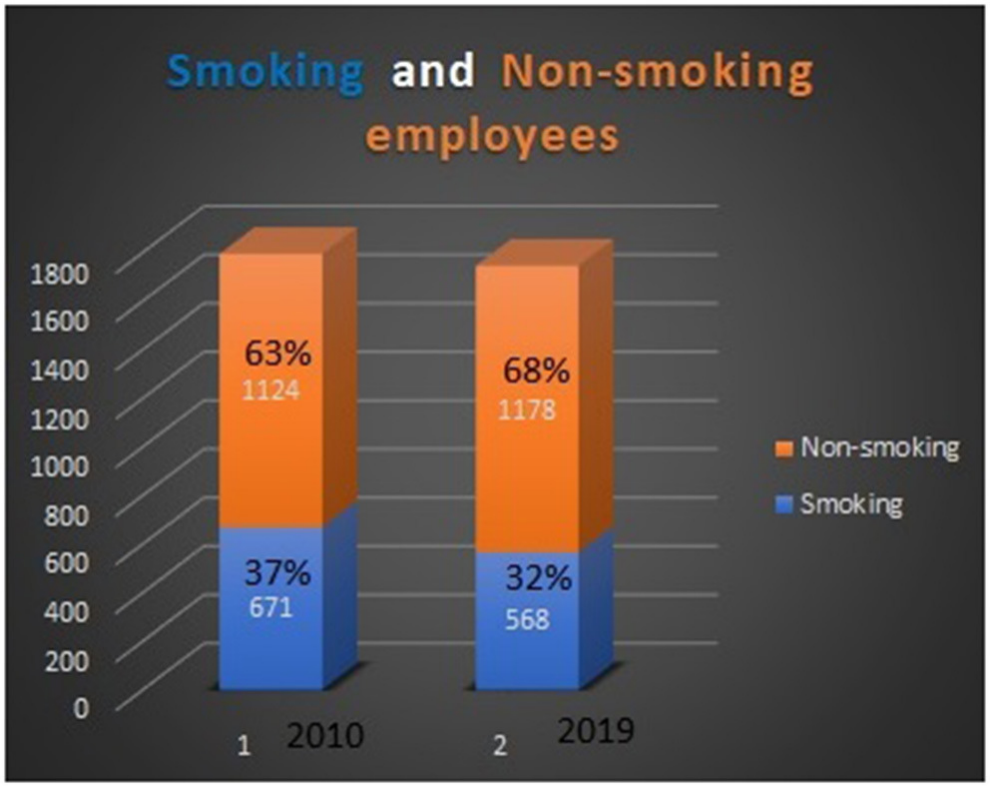
Smoking vs. non-smoking employees in 2010 and 2019.

**Figure 3:**
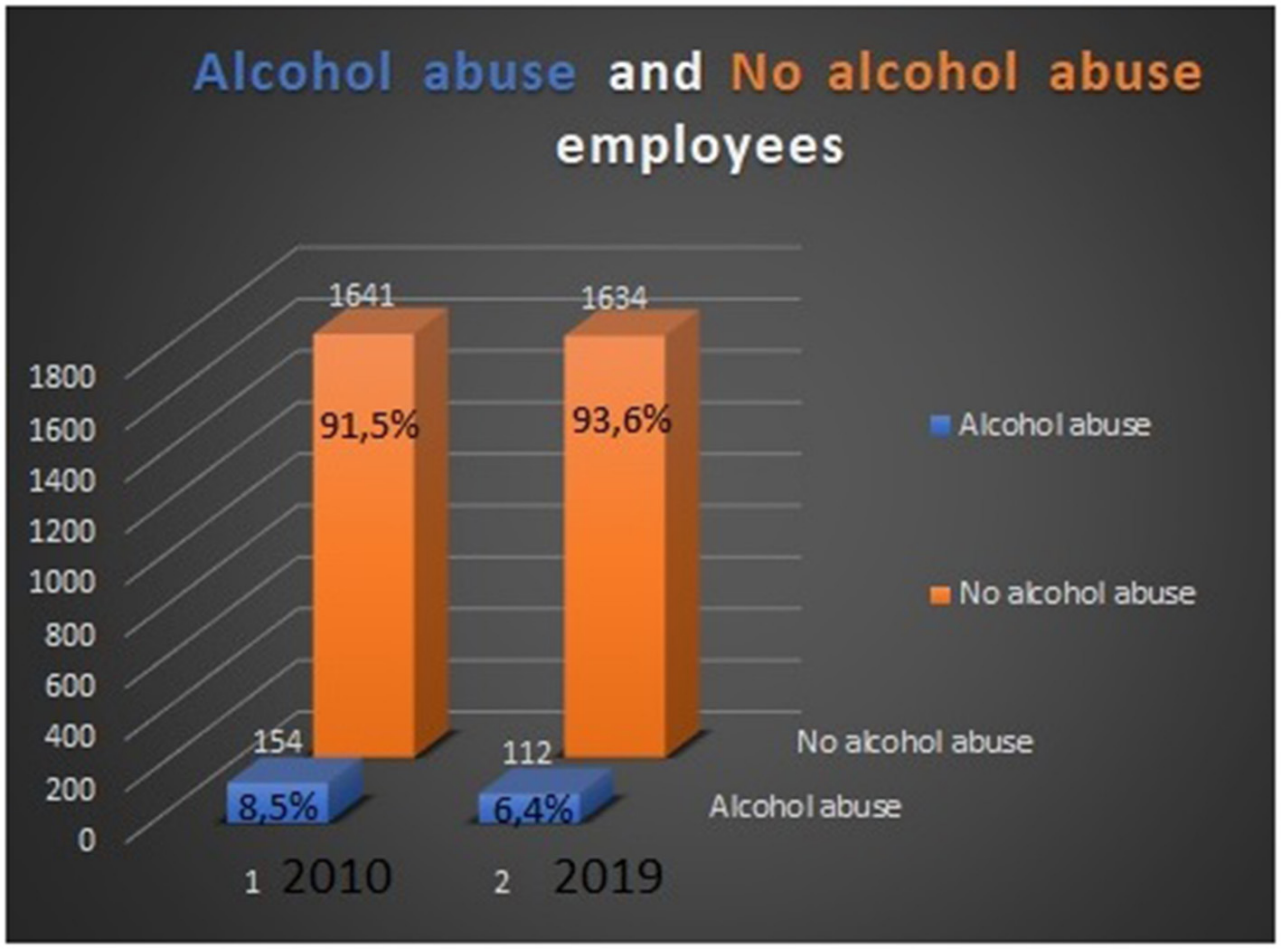
Alcohol abuse vs. no-alcohol abuse employees in 2010 and 2019.

The following activities complement our programs in workplace health promotion for employees and students:

- Quitting smoking (see Figure 2).
- Giving up excessive alcohol consumption (see Figure 3).
- Alimentation advisership necessary for all employees.
- Employees access to healthy meals in the cafeteria or university restaurant, buffet, and food and drink vending machines, which are controlled regularly by the occupational health specialist.
- Stress–prevention and stress–handling trainings (6) in the form of individual or group training.
- Physical exercise, certain home-based physical activities, medical gymnastics, yoga, and swimming.

The swimming pool on campus can be used by all employees at 50% reduced entrance fee. Those over 40 years of age or suffering of chronic diseases pay no entrance fee for aquatic gymnastics. We complement these activities with games (football, handball, volleyball, etc.), fitness facilities, and organize excursions and as a special recreation form, offer dance activities as well. Any of these activities are available to all employees and are recommended depending on the individual's disease, physical condition, age, risk factors, and motivation.

**Figure 4:**
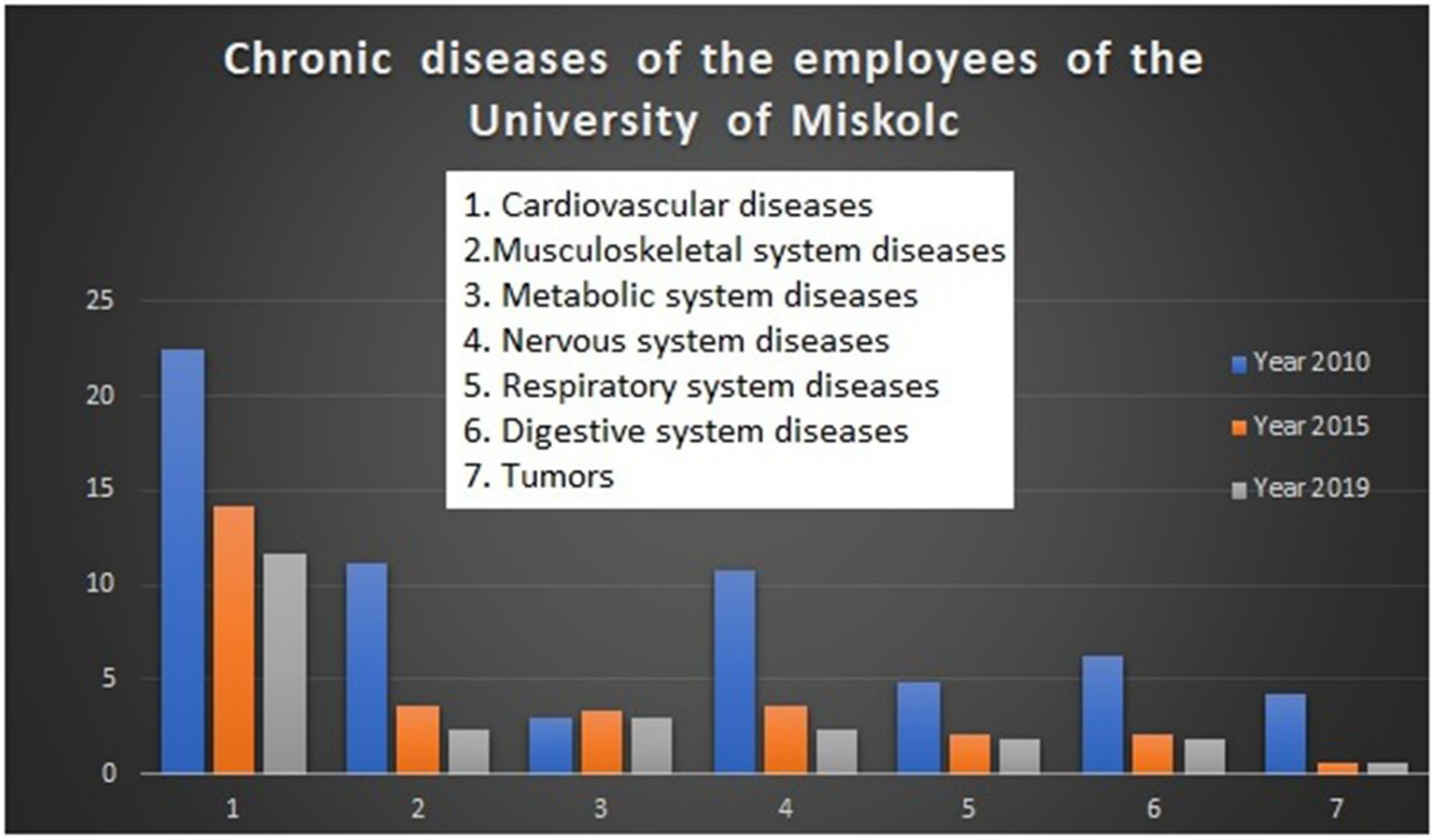
Decrease of the incidence of chronic diseases of employees of the university.

The efficiency of this complex program is well illustrated in Figure 4.

## Education

Health promotion is an optional subject for all faculties and compulsory for the programs of the Faculty of Heath Sciences. We offer individual or group advisership, publish periodical articles on the subject, deliver lectures, produce videos, and publish online materials.

We had only 86 COVID-19 infections at the University. There were no cases of occupational disease.

We would like to develop further the Center and expand our programs in the spirit of a Hindu proverb, which says:

“The greatest thing is not to be better than others, but better than yourself.”

Closer to home, László Németh, a famous Hungarian writer said:

“The real strength of a society lies not in its rocket-like talents, but in the values of ordinary people working at ordinary tasks in society…”

Our never-ending task is to preserve that value.

## Research

The actors in the world of work may have a different view of employees' health. Surveys and research are needed to reveal the situation of occupational health and safety from the perspective of the actors of the world of work in the same workplace, seeking the opinions of employers, workers, and those who ensure occupational health and safety.

### “Health and Safety at Work” Research Project Series

The objectives of the project series included sector-specific situational awareness and knowledge expansion in 11 industry sectors to improve the working conditions in the given sectors, the analysis of knowledge identified and the exploitation of the research results that have emerged.

The main objectives of the project applications were promoted at the national level (Széchenyi 2020 and GINOP) and community level (EU2020). As a result, the target objectives were set out in the Operational Program for Economic Development and Innovation.

The novelty of the research is that in Hungary the situation has not yet been revealed in the same workplace at such a wide scale and at such depth (competences, tools, quality)—involving employers, employees, occupational healthcare workers, occupational safety and advocacy groups (trade unions, associations representing employers).

In particular, the research involved risk assessment (ergonomic, psychosocial factors, aging workers), job aptitude tests and occupational health promotion in 11 sectors (rubber industry, transport, retail, wholesale, education, healthcare, telecommunication, financial services, wood industry, food industry, and water supply), and the research analysis based on the questionnaires that covered over 2,500 pages, which were published on the web pages of the given sectors, see for example, Refs. (7, 8).

We have explored development opportunities in this area to significantly increase the efficiency of occupational health and safety, and to explore opportunities to make the work of the representatives of the involved groups even more effective.

### Analysis of Questionnaires

We used general questionnaires to determine the headcount figures, the state of the occupational safety, risk factors, as well as for occupational health promotion, and company health policy, etc. on the state of occupational health and safety based on the opinions of all four main actors in the world of work.

### High Risk Areas

Ergonomic questionnaires were used to assess the typical risks related to specific areas (shift-standing, driving, industrial work, office work), employees (we formed subgroups of employees,—they received a separate questionnaire, to avoid repetitions and the questionnaires became more manageable), and employers (we requested answers on how the employer handles these risks). The occupational health and safety professionals were asked for a response on how they performed their professional tasks in this field.

Questionnaires for aging workers were used to assess age-related risks for workers over 45 years of age. Employers, occupational health specialists, and occupational safety professionals were asked about these risks. As for the assessment of psychosocial risks, and considering the data protection, we have asked only the occupational health specialists.

The research analysis contains two parts as follows:

**Part one:** This describes the history of the field, international and national statistical indicators, the characteristics of work and the work environment, typical occupations, and risk factors.

**Part two:** This contains the results of statistical evaluations (in text and figures), and commentary on the results and assessment of the situation. The latter follows the steps of SWOT analysis, the acronym stands for: Strengths, Weaknesses, Opportunities and Threats.

We formulated recommendations to eliminate weaknesses, make better use of opportunities and counter threats.

### SWOT Analysis

#### Strengths

The interest representatives (Occupational Safety and Health, OSH representatives) are present in 100% of cases. The risk analysis is done in 100%. The occupational health and safety at work professionals are present in 100%. The occupational health promotion is organized in 45% of cases.

#### Weaknesses

The employers do not express their views in all cases. Health and safety at work is often considered an “administrative burden.” A comprehensive OSH strategy is often lacking. “Elderly-friendly workplaces” appear only in 9% of cases.

#### Opportunities

The weaknesses identified by the SWOT analysis draw attention to the need for introducing corrective procedures so that these can be converted into strengths. In addition to measures, OSH representatives and employees' OSH education is also beneficial.

#### Threats

Unforeseen events, as restrictions due to the COVID-19 pandemic, can disrupt planned activities, and delay the scheduled tasks.

### Conclusions of the Research Series

Cooperation with the professionals, interest representatives—even in areas that have not yet been exploited, such as research—can effectively serve the interests of employees (maintaining their health). At the same time, they will have access to data, which is also a serious argument for maintaining the health of workers in negotiations with employers and legislators (more data, more arguments). It is also a great opportunity for education, fulfilling the need for a change in attitude.

It is necessary to revise the legislation to eliminate contradictions—to create a uniform occupational health law (9), to strengthen OSH inspectorates to control employers, and a closer cooperation of interest representatives with the profession (occupational health and job security). The goal of increasing employment by creating competitive jobs will be achieved by highlighting the importance of working conditions that improve attitudes leading to this and by pointing out health and safety aspects, as well as by supportive attitudes toward employees and employers.

On the second level of the target employers and employees, the major factors in the world of work need the advocacy of organizations to improve working conditions, the activity of occupational health and safety professionals.

As other contributions at the lowest level of the target pyramid, the projects were focusing on operational objectives, taking into account the territorial scope of the projects (South Great Plain, South Transdanubia, Northern Great Plain) and the development of sector-specific activities (aiming to improve working conditions) through the use of newly developed OSH tools for this purpose.

## International Impact

The activity of the Occupational Health and Methodology Center of the University of Miskolc presented in several international conferences became a starting point for a larger international cooperation in the framework of a Know-How Exchange Program (KEP) Improving Occupational Health and Safety System in Republic of Moldova, co-funded by the Central European Initiative, (PROJECT REFERENCE KEP/Ref: No. 304.4.22-20) involving three countries and their universities. The project partners are the Department of Public Health and Forensic Medicine of the University of Pavia from Italy and the Center of Healthcare and Methodology of the University of Miskolc from Hungary, acting as knowledge providers, and Nicolae Testemitanu State University of Medicine and Pharmacy from Republic of Moldova, as knowledge beneficiary.

The project is aimed to share the experience of the large-scale research conducted in Hungary to prepare the improvement the Occupational Health and Safety in Moldova.

The specific objectives of the project are as follows: “(1) To implement the most appropriate research methodologies for estimating risk factors due to occupational exposures; (2) To review the national legislative and normative acts in order to facilitate the ratification of ILO Convention No. 161 on occupational health and safety in the Republic of Moldova; (3) To strengthen the competencies of the academic staff in occupational health and safety; (4) To establish a sustainable research partnership between the Republic of Moldova, Italy and Hungary, with coordination and harmonization of the modernized occupational exposure assessment methods, and the facilitation of the integrated research strategy on occupational health” (10).

## Data Availability Statement

The original contributions presented in the study are included in the article/supplementary material, further inquiries can be directed to the corresponding author/s.

## Author Contributions

SF wrote all sections of the manuscript.

## Funding

This research was supported by the Know-How Exchange Program (KEP) Improving Occupational Health and Safety System in Republic of Moldova, co-funded by the Central European Initiative, (PROJECT REFERENCE KEP/Ref: No. 304.4.22-20) involving three project partners: Department of Public Health and Forensic Medicine of the University of Pavia from Italy and the Centre of Healthcare and Methodology of the University of Miskolc from Hungary, acting as knowledge providers, and Nicolae Testemitanu State University of Medicine and Pharmacy from Republic of Moldova, as knowledge beneficiary.

## Conflict of Interest

The author declares that the research was conducted in the absence of any commercial or financial relationships that could be construed as a potential conflict of interest.

## Publisher's Note

All claims expressed in this article are solely those of the authors and do not necessarily represent those of their affiliated organizations, or those of the publisher, the editors and the reviewers. Any product that may be evaluated in this article, or claim that may be made by its manufacturer, is not guaranteed or endorsed by the publisher.
